# Circadian Clock Genes *Per1* and *Per2* Regulate the Response of Metabolism-Associated Transcripts to Sleep Disruption

**DOI:** 10.1371/journal.pone.0052983

**Published:** 2012-12-28

**Authors:** Jana Husse, Sophie Charlotte Hintze, Gregor Eichele, Hendrik Lehnert, Henrik Oster

**Affiliations:** 1 Max Planck Institute for Biophysical Chemistry, Göttingen, Germany; 2 Medical Department I, University of Lübeck, Lübeck, Germany; University of Alabama at Birmingham, United States of America

## Abstract

Human and animal studies demonstrate that short sleep or poor sleep quality, e.g. in night shift workers, promote the development of obesity and diabetes. Effects of sleep disruption on glucose homeostasis and liver physiology are well documented. However, changes in adipokine levels after sleep disruption suggest that adipocytes might be another important peripheral target of sleep. Circadian clocks regulate metabolic homeostasis and clock disruption can result in obesity and the metabolic syndrome. The finding that sleep and clock disruption have very similar metabolic effects prompted us to ask whether the circadian clock machinery may mediate the metabolic consequences of sleep disruption. To test this we analyzed energy homeostasis and adipocyte transcriptome regulation in a mouse model of shift work, in which we prevented mice from sleeping during the first six hours of their normal inactive phase for five consecutive days (*timed sleep restriction* – TSR). We compared the effects of TSR between wild-type and *Per1/2* double mutant mice with the prediction that the absence of a circadian clock in *Per1/2* mutants would result in a blunted metabolic response to TSR. In wild-types, TSR induces significant transcriptional reprogramming of white adipose tissue, suggestive of increased lipogenesis, together with increased secretion of the adipokine leptin and increased food intake, hallmarks of obesity and associated leptin resistance. Some of these changes persist for at least one week after the end of TSR, indicating that even short episodes of sleep disruption can induce prolonged physiological impairments. In contrast, *Per1/2* deficient mice show blunted effects of TSR on food intake, leptin levels and adipose transcription. We conclude that the absence of a functional clock in *Per1/2* double mutants protects these mice from TSR-induced metabolic reprogramming, suggesting a role of the circadian timing system in regulating the physiological effects of sleep disruption.

## Introduction

The prevalence of obesity has dramatically increased in most industrialized countries within the last decades [1]. At the same time, average sleep times have decreased. Whereas self-reported daily sleep duration was eight to nine hours in 1960, it was close to seven hours in 1995 [2], [3]; today, almost a third of adults report sleeping less than six hours per night [4]. Epidemiological studies have shown that short sleep is associated with higher body mass index (BMI) [5], indicating that sleep curtailment might promote obesity. In line with this, shift work, which is often accompanied by severe disruption of normal diurnal sleep patterns and reduction of overall sleep quality [6], is associated with a higher risk of developing obesity, type-2 diabetes and metabolic syndrome [7], [8], [9], [10], [11]. A causal link between sleep disruption and metabolic impairments has been established in a number of laboratory studies (reviewed in [12], [13]). Sleep restricted humans show increased appetite and – if allowed free access to food – eat more [14], [15]. In addition, circulating levels of metabolically relevant hormones such as leptin and ghrelin are altered and pre-diabetic changes in glucose homeostasis are observed (reviewed in [13], [16]). Interestingly, the blood levels of several adipokine hormones correlate with sleep duration [17], suggesting that (white) adipose tissue may be an important peripheral target of sleep loss [18]. In line with this idea, sleep loss or poor sleep quality can lead to dyslipidemia and increased abdominal fat accumulation in humans [7], [11], [19], [20]. Rodent models of sleep restriction confirmed metabolic effects on glucose homeostasis, and changes in liver physiology and hepatic transcription have been described in some detail [21], [22], [23]. In addition alterations in plasma levels of adipokines were shown in rodents [24]. However, the effects of sleep disruption on adipocyte function remain largely unknown.

There is accumulating evidence that the circadian timing system is tightly linked to metabolic regulation. Many key enzymes in metabolically relevant tissues like liver, adipose tissues or pancreas are clock controlled. Clock disruption by genetic or behavioral means can result in severe metabolic impairments including obesity, insulin resistance and metabolic syndrome (reviewed in [25], [26], [27]). Thus sleep and circadian disruption appear to have very similar metabolic endpoints, which led us to hypothesize that the circadian clock might mediate the metabolic effects of sleep disruption. To test this we compared the effects of sleep disruption between wild-type and clock-deficient mice. We used a genetic model of clock deficiency, in which both *Period* genes (*Per1* and *Per2*) are disrupted [28], [29]. *Per1* and *Per2* are key players in the molecular clock mechanism and simultaneous deletion of *Per1* and *Per2* in mice destroys the functionality of the clock and abrogates circadian behavioral and physiological rhythms [29]. Following our hypothesis that the circadian clock might mediate the effects of sleep disruption, we predicted a blunted response to sleep disruption in clock-deficient *Per1/2* mutants.

Our study uses a mouse model of shift work, in which mice were prevented from sleeping during the first six hours of their normal inactive phase for five consecutive days (termed *timed sleep restriction* – TSR). We show that this protocol results in increased food intake, hyperleptinemia and body weight changes in wild-type mice. In addition we observe a significant transcriptional reprogramming of white adipose tissue, indicative of increased lipogenesis. Some of these effects persist for at least one week after the end of TSR. In contrast, clock-deficient *Per1/2* double mutant mice show blunted metabolic effects of TSR on most parameters. Thus the absence of a functional clock appears to protect these mice from the TSR-induced effects, suggesting that the circadian clock at least partly mediates the metabolic effects of sleep disruption.

## Materials and Methods

### Animals

All animal experiments were carried out in compliance with the German Law on Animal Welfare and were approved by the Office for Consumer Protection and Food Safety of the State of Lower Saxony. *Per1/2* double mutant mice were generated from *Per1* (*Per1^Brd1^*) [29] and *Per2* (*Per2^Brd1^*) [28] mutant mice (both backcrossed to C57BL/6 for at least ten generations). Genotyping was performed as described [28], [29]. 9–11 week old male C57BL/6 and congenic *Per1/2* mice were used for all experiments. Body weight at the beginning of the experiment was not different between wild-type and *Per1/*2 mutant mice.

### Food and body weight measurements

Animals were group-housed during the experiments (4–6 mice per cage). Body weight was measured at the same time of the day once per week under each condition (on the last day of each condition) and body weight gain/day was calculated by dividing the weekly body weight gain by 7. Food intake was measured during a 24 hour window during control conditions, on the last day of TSR and on the 7^th^ day during recovery. A daily food intake value was obtained for each cage and the daily food intake/mouse was calculated by dividing the whole-cage food intake by the number of mice in this cage. Thus each food intake value represents already an average over 4–6 mice.

### Timed sleep restriction

Timed sleep restriction (TSR) was performed using a gentle handling protocol during the first six hours of the light phase (*Zeitgeber* time (ZT) 0–6) for five consecutive days. This gentle handling approach is an established protocol for the disruption of diurnal sleep patterns in mice [30], [31], [32] and rats [33]. Mice were observed throughout the TSR period by an experimenter. As soon as one of the animals adopted a sleeping posture (eyes closed, no movement for more than five seconds), they were woken up by introducing novel and interesting objects into the cage or by gently touching the animal with a plastic pipette. We counted the number of interventions necessary to keep wild-type and *Per1/2* mutant mice awake at different hours of TSR and found that a maximum of nine interventions per hour were required (Fig. S1). Together with the maximum of five seconds that an animal was allowed in a sleep-like posture, this means that during the six hours of sleep restriction mice were maximally allowed 4.5 minutes of sleep (1.2% of the TSR time), equaling 1.9% of the sleep time of a normal undisturbed C57BL/6 wild-type mouse during that time [34], [35]. Of note, mice were undisturbed during the rest of the day (ZT6–18) and allowed to compensate for any sleep lost in the morning. Mice were kept in grouped cages (3–5) under 12 h∶12 h light-dark conditions (LD) with 50 lux light intensity in the light phase, constant temperature (20±0.5°C) and humidity (50–60%), and *ad libitum* access to standard chow food (Ssniff V1126, Soest, Germany) and water. Activity was measured using custom-made passive infrared sensors installed on the lid of each cage and analyzed using ClockLab Software (Actimetrics, Evanston, IL). Animals were assigned to the control (no TSR), the timed sleep restriction (TSR for five consecutive days), or the recovery group (TSR followed by one week of undisturbed sleep). All animals were sacrificed by cervical dislocation (under dim red light during the dark period). Trunk blood was collected into EDTA-coated capillary tubes (Microvette CB300, Sarstedt, Nümbrecht, Germany) to obtain plasma after centrifugation.

### Plasma metabolite determination

Plasma glucose levels were determined using a blood glucose meter (Accu-Chek Aviva, Roche, Mannheim, Germany). Plasma glycerol and triglyceride levels were measured using the Serum Triglyceride Determination Kit (Sigma, St. Louis, MO) and non-esterified free fatty acid (NEFA) levels were measured using the Serum/Plasma Fatty Acid Kit (Zenbio, Durham, NC) according to the manufacturers' protocols.

### Immunoassays

Corticosterone was measured from plasma samples using the ImmuChem Double Antibody 125I-Radioimmunoassay Kit (MP Biomedicals, Solon, OH); plasma leptin was measured using the Mouse Leptin ELISA Kit (Crystal Chem, Downers Grove, IL) according to the manufacturers' protocols.

### RNA isolation and quantitative real-time PCR (qPCR)

Total RNA was extracted from RNAlater- (Ambion, Darmstadt, Germany) protected epididymal adipose tissue samples collected in the middle of the dark phase (ZT18) using RNeasy Lipid Tissue Mini Kit (Qiagen, Hilden, Germany). On-column DNase treatment was performed to avoid residual genomic DNA contamination. cDNA was synthesized using Superscript II reverse transcriptase (Invitrogen, Darmstadt, Germany) and Oligo-dT primers, followed by quantitative real-time (q)PCR using iQ SYBR Green Supermix on an iCycler thermocycler (Bio-Rad, Munich, Germany) according to the manufacturer's protocol. Primer sequences are available on request. *Eef1α* expression was used for normalization and relative quantification (ΔΔCT method) was performed as described [36].

### Microarray analysis

RNA was extracted as described above; cRNA synthesis and labeling was performed by the microarray core facility of the University of Göttingen according to standard protocols. cRNA was hybridized to Affymetrix GeneChip Mouse Genome 430A 2.0 arrays and raw fluorescent intensity values were normalized using the MAS5 algorithm and Affymetrix Expression Console software. Only transcripts with significant expression calls were included for further analysis. To test for significant regulation between control and TSR conditions we used Student's t-tests with Benjamini-Hochberg correction for multiple testing to control for false discovery rate [37]. Only regulated genes (false discovery-corrected p-value<0.05), which were at least 2-fold up or down regulated were used for further analysis. Gene ontology enrichment analysis was performed using the Cytoscape plug-in for Bingo [38]. As reference for ontology analysis a list of all genes, which were expressed in WAT at ZT 18 (genes with significant expression call) was used. Results were visualized using GoSurfer [39]. Heat maps of log-transformed normalized values were constructed using Dchip software [40]. Raw data have been deposited in the GEO database (www.ncbi.nlm.nih.gov/geo/), accession number is GSE38921.

### Data analysis

All data are shown as mean +/− SEM. Statistical comparisons were made using GraphPad Prism (GraphPad, La Jolla, CA) or Excel software (Microsoft, Redmond, WA); p-values below 0.05 were considered significant. Student's t-tests were used for comparison of two groups and one-way ANOVAs with Bonferroni post-tests when more than two groups were compared. Where time and group interactions or genotype and group interactions were compared, two-way ANOVAs with Bonferroni post-tests were used. Unless stated otherwise statistical comparisons were always made between control and TSR or control and recovery period groups for the same genotype.

## Results

### Timed sleep restriction (TSR) alters diurnal activity profiles, food intake and leptin levels in wild-type mice

**Figure 1:**
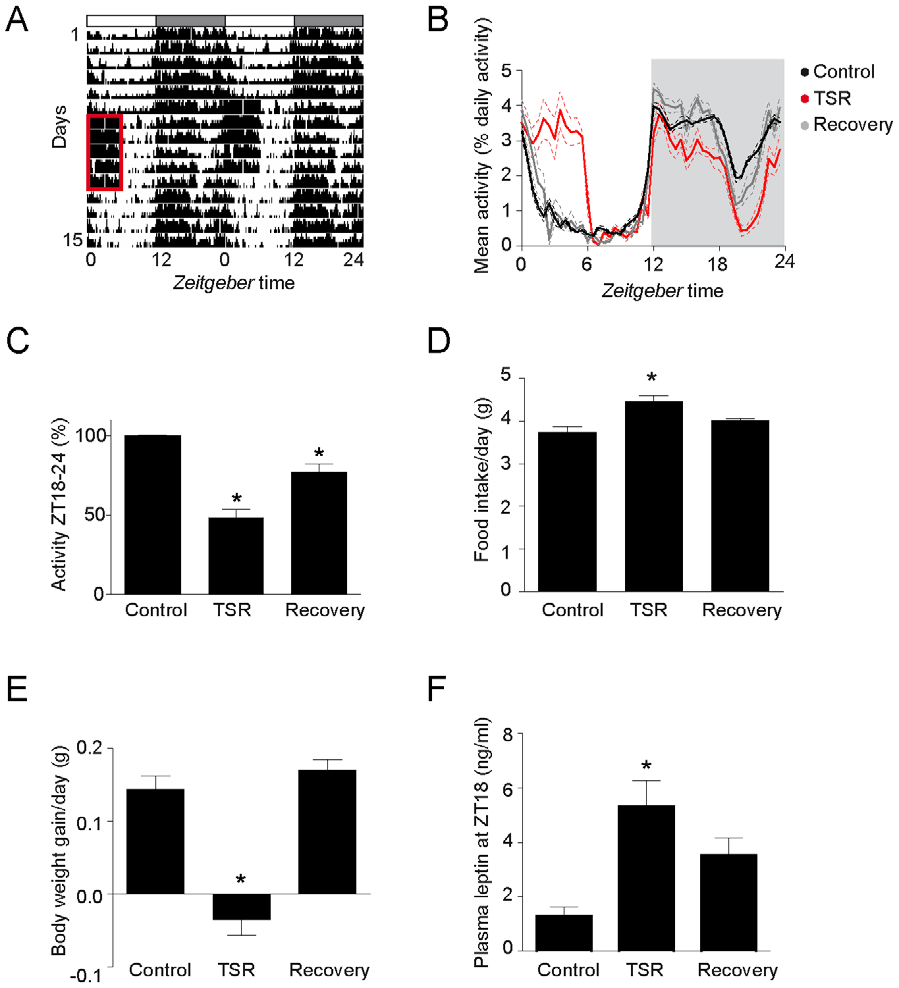
Timed sleep restriction (TSR) alters diurnal activity profiles, food intake and plasma leptin levels in wild-type mice. A) Representative double plotted activity recording during five days of control, five days of TSR and five days of recovery. TSR (ZT0–6) is highlighted by a red rectangle. Light and dark phases are indicated by white and grey boxes, respectively. B) Mean diurnal activity profiles were generated by plotting the relative locomotor activity for every 30 min bin as percentage of total daily activity. Light and dark phases are indicated in white and grey, respectively. Data are plotted as mean ± SEM (dotted lines). C) Relative activity during the second half of the night (ZT 18–24). *: p<0.001 control vs. TSR, p<0.05 control vs. recovery, one-way ANOVA with Bonferroni post-test. D) Food intake during one day of control, during the last day of TSR and during the 7^th^ day of recovery. * p<0.001 control vs. TSR, one-way ANOVA with Bonferroni post-test. P<0.001 control vs. TSR, p<0.01 control vs. recovery, t-test. E) Body weight gain per day of control, TSR and recovery. *: p<0.001 control vs. TSR, one-way ANOVA with Bonferroni post-test. F) Plasma leptin levels at ZT18 measured on one day of control, the last day of TSR and the 7^th^ day of recovery. *: p<0.05 control vs. TSR, one-way ANOVA with Bonferroni post-test. P<0.01 control vs. TSR, p<0.05 control vs. recovery, t-test. All data are shown as mean ± SEM. Sample sizes were 5 per group for activity, 3–8 per group for food intake, 17–33 per group for body weight and 4–5 per group for leptin data.

We subjected wild-type mice to TSR by preventing them from sleeping in the first six hours of their normal inactive phase (*Zeitgeber* time (ZT) 0–6) using a gentle handling protocol. This protocol was performed for five consecutive days, mimicking a five-day night shift schedule. Because stress might confound metabolism-associated readouts, we tested the effects of TSR on stress axis regulation by measuring plasma corticosterone levels every six hours during the last day of TSR. Diurnal corticosterone profiles were not significantly changed by five days of TSR (Fig. S2; but see phase shifts observed after ten days – [22]), indicating a relatively stress-free procedure. To investigate the effects of TSR on diurnal activity profiles, we measured locomotor activity during control conditions, TSR and during one week after the end of TSR (recovery) (Fig. 1A–C). We compared relative activity values for 6 hour bins over the course of the day. As expected, activity was strongly increased between ZT0–6, the time of gentle handling (Fig. S3A). During the times where the animals were left undisturbed (ZT6–24), the most pronounced effect on activity levels was observed between ZT18–24 (Fig. S3B,C; Fig. 1C). At this time activity levels were strongly decreased during TSR. Interestingly, the diurnal activity profiles were only partially restored during recovery with mice retaining decreased activity levels in the second half of the dark period (Fig. 1A–C). Acute disruption of diurnal activity patterns has been described in other mouse models of shift work [22], [41], [42] and both the acute and prolonged effects of activity we describe are reminiscent of what has been observed in human shift workers [43], indicating that our TSR procedure mimics human shift work conditions. Despite this pronounced change of diurnal activity patterns during TSR and recovery, total activity levels were unaltered (Fig. S3D).

Next we investigated effects of TSR associated with energy metabolism. In line with what has been reported earlier for sleep-deprived humans [15] and rodents [44], [45], [46], food intake was increased during TSR (Fig. 1D). However in contrast to many other rodent studies, we did not only investigate acute but also prolonged effects of TSR (recovery). Surprisingly, there was a clear trend towards persistence of hyperphagy during recovery (Fig. 1D). As reported before [44], [45], [46] mice did not gain body weight during the week of TSR. However there was a trend towards increased body weight gain during recovery compared to control conditions (Fig. 1E). This finding is in line with the hyperphagy observed during TSR and could be an early indicator of deregulated energy homeostasis.

As the anorexigenic adipokine leptin had previously been shown to be altered after sleep restriction in humans [14], [47], [48] and rodents [22], [23], we measured plasma leptin during control, TSR and recovery conditions. To avoid acute effects of TSR-associated manipulations we determined leptin levels at the opposite phase of the LD cycle in the middle of the night (ZT18). At this time plasma leptin was elevated more than 3-fold after TSR in wild-type mice (Fig. 1F). During recovery, leptin appeared still increased, but this effect only reached significance in a pair-wise test (Fig. 1F). In summary, in wild-type mice TSR results in disrupted activity profiles accompanied by hyperphagy and hyperleptinemia. Some of these effects persist for at least one week after the end of TSR.

### TSR alters diurnal activity profiles, but not food intake or leptin levels in *Per1/2* mutants

**Figure 2:**
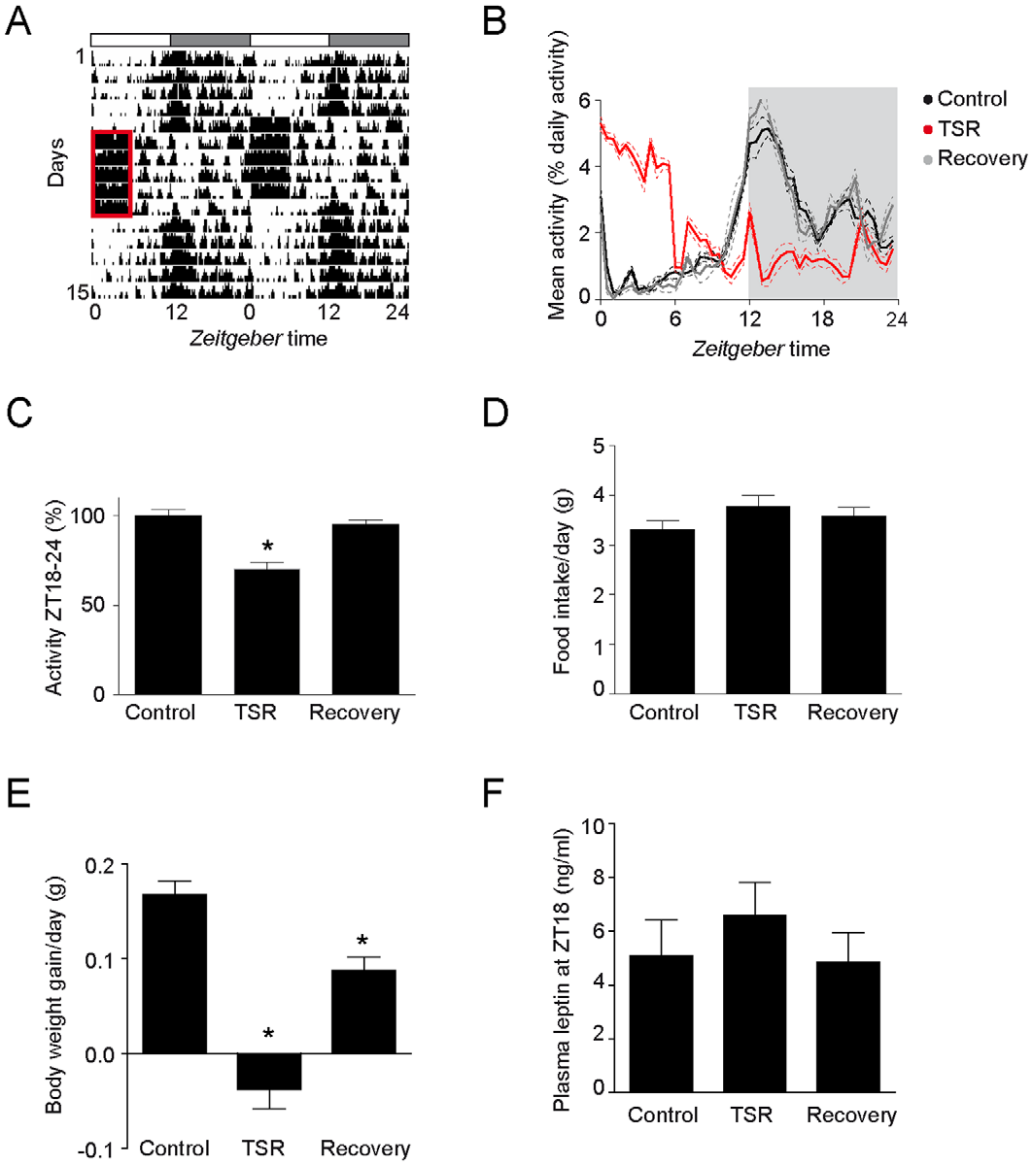
TSR alters diurnal activity profiles, but not food intake or plasma leptin levels in *Per1/2* mutants. A) Representative double plotted activity recording during five days of control, five days of TSR and five days of recovery. TSR (ZT0–6) is highlighted by a red rectangle. Light and dark phases are indicated by white and grey boxes, respectively. B) Mean diurnal activity profiles (n = 5) were generated by plotting the relative locomotor activity for every 30 min bin as percentage of total daily activity. Light and dark phases are indicated in white and grey, respectively. Data are plotted as mean ± SEM (dotted lines). C) Relative activity during the second half of the night (ZT 18–24). Activity is expressed relative to the average activity during the same time in the control week (in %). *: p<0.001 control vs. TSR, two-way ANOVA with Bonferroni post-test, see also Suppl. Table ST1. D) Food intake during one day of control, during the last day of TSR and during the 7^th^ day of recovery. Two-way ANOVA with Bonferroni post-test not significant, see also Table S1. E) Body weight gain per day of control, TSR and recovery. *: p<0.001 control vs. TSR, p<0.05 control vs. recovery, two-way ANOVA with Bonferroni post-test, see also Suppl. Table ST1. F) Plasma leptin levels at ZT18 measured on one day of control, the last day of TSR and the 7^th^ day of recovery. Two-way ANOVA with Bonferroni post-test, not significant, see also Table S1. All data are shown as mean ± SEM. Sample sizes were 5 per group for activity, 4–8 per group for food intake, 17–33 per group for body weight and 4–5 per group for leptin.

We next wanted to test our hypothesis that some of the effects of sleep disruption might be mediated by the circadian clock. We subjected circadian clock-deficient *Per1/2* double mutant mice to the same TSR procedure with the prediction that the clock-deficiency in these mutants should result in reduced TSR effects. In contrast to wild-type mice, TSR in *Per1/2* mutants resulted in a loss of the normal LD locomotor activity pattern (Fig. 2A, B). During TSR, their main activity period was shifted to the early light phase and, consequently, the distribution of activity between light and dark phases was reversed. Comparable to what we had observed in wild-type mice, the activity of *Per1/2* mutants in the second half of the dark phase (ZT18–24) was reduced during TSR (Fig. 2C, Table S1). However, this effect appeared to be an acute effect of the TSR procedure, because a rapid restoration of activity patterns similar to control conditions was observed during recovery (Fig. 2B,C, Table S1). Despite clear effects on activity profiles, the responses to TSR associated with energy metabolism were severely blunted in *Per1/2* mutants. Food intake was not changed by TSR, as were plasma leptin levels at ZT18 (Fig. 2D,F, Table S1). Of note, baseline levels of leptin were increased in *Per1/2* mutants compared to wild-type baseline levels (Fig. 2F, Table S1), which is in accordance with earlier studies [49]. The acute effects on body weight gain during TSR were similar to wild-type mice (Fig. 2E, Table S1). However, *Per1/2* mutants did not show increased body weight gain in the recovery week, but instead weight gain remained significantly below control conditions (Fig. 2E, Table S1). Two-factor analysis revealed mean effects of TSR and genotype on the described parameters (Table S1). Of note, some of the parameters (body weight and activity) revealed significant interaction effects, confirming that *Per1/2* mutants react differently to TSR than wild-type mice. In summary, *Per1/2* mutant mice showed strong responses to TSR at the behavioral level, while at the same time they appeared to be protected from the physiological effects of TSR.

### TSR-induced transcriptional reprogramming in white adipose tissue (WAT) in wild-type mice

**Figure 3:**
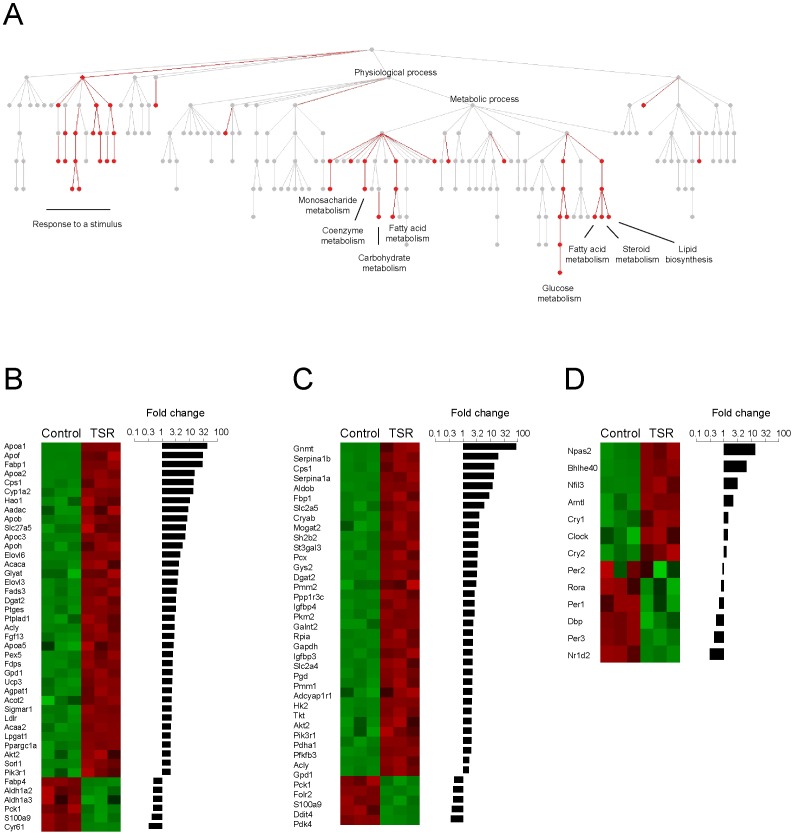
TSR-induced transcriptional reprogramming of WAT in wild-type mice. A) Gene ontology analysis of microarray data of epididymal WAT at ZT18 on the last day of TSR compared to control WAT at ZT18. Only nodes (GO categories) with at least 10 regulated genes are shown. Significant overrepresentation of nodes is highlighted in red. B) Individual normalized log-transformed expression values for all genes involved in lipid metabolic pathways which are regulated at least 2 fold between control and TSR are plotted sorted for fold change. C) Individual normalized log-transformed expression values for all genes involved in glucose metabolic pathways which are regulated at least 2 fold between control and TSR are plotted ordered by fold change. D) Clock gene regulation by TSR in WAT at ZT18 sorted for fold change. Only clock genes with significant expression under control conditions are shown. With the exception of *Per2* and *Rora*, all clock genes were significantly regulated by TSR. Green represents low expression, red represents high expression. Sample sizes were 3 per group.

TSR resulted in altered leptin levels in wild-type mice indicating a deregulation of adipose physiology, in line with previous findings from sleep restricted humans [47] and human shift workers [50], [51]. We observed similar changes in the expression levels of *leptin* mRNA in white adipose tissue (Fig. S4), which prompted us to analyze the underlying transcriptome changes. We performed microarray analyses comparing WAT transcriptome regulation at ZT18 between control and TSR mice. 25% of all expressed genes were significantly up or down regulated by TSR with 3.2% being regulated more than 2-fold (Table S2). Gene ontology analysis revealed an over-representation of metabolic processes (Fig. 3A). Most strikingly, both lipid (44 of 631 genes) and carbohydrate metabolism associated transcripts (39 of 411 genes) were significantly over-represented (and several genes of other metabolic pathways were also affected), suggesting a transcriptional reprogramming of key WAT metabolic pathways (Fig. 3B, C). Interestingly, most of these transcripts were up regulated and only a few showed decreased expression levels during TSR. Among the up regulated genes we found a number of key metabolic enzymes involved in lipid biosynthesis (*Acaca*, *Agpat1*, *Dgat2*, *Mogat2*) as well as glycolysis (*Slc2a4*, *HK2*, *Pfkfb3*, *Gapdh*), indicative of increased lipogenesis. Interestingly we found that among the clock genes, which were expressed in WAT at ZT18, 11 out of 13 were significantly regulated by TSR (only *Per2* and *Rora* were not significantly changed; Fig. 3D). Some of those showed strong changes (e.g. *Npas2* was almost 14-fold up-regulated), suggesting pronounced TSR effects on the WAT clock.

### Prolonged and wild-type-specific induction of lipogenic and glycolytic genes in WAT by TSR

**Figure 4:**
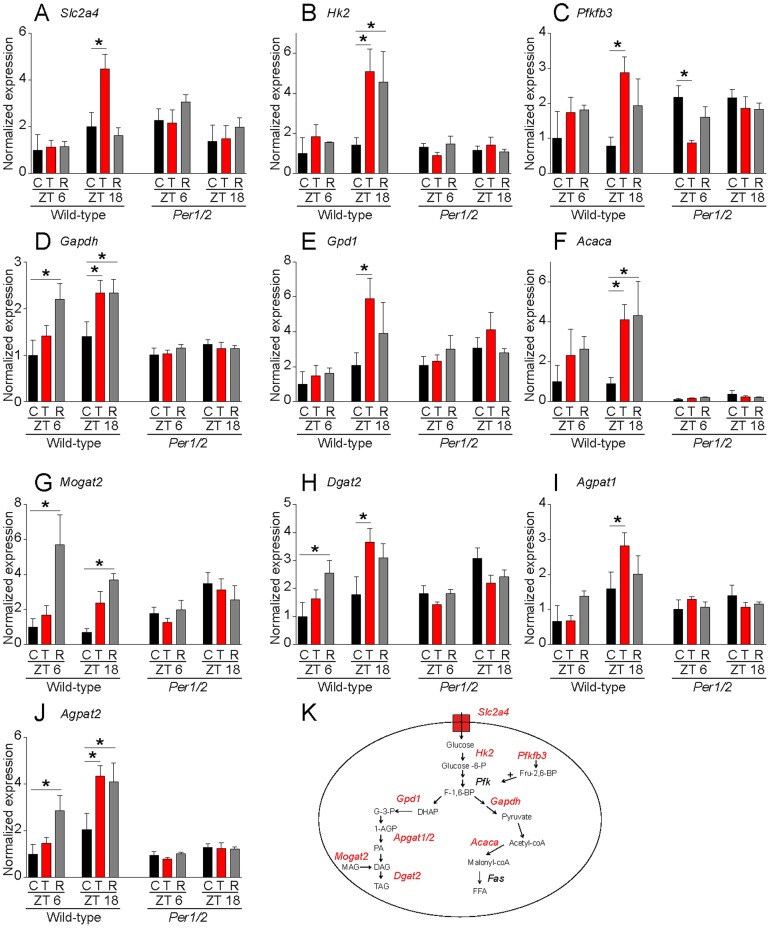
Sustained and wild-type-specific induction of lipogenic and glycolytic genes in WAT. A–J) Expression of glycolytic and lipogenic genes in epididymal WAT at ZT6 and at ZT18 in control conditions, on the last day of TSR and on the 7^th^ day of recovery for wild-type and *Per1/2* mutant animals. Expression values are normalized to the mean of the wild-type control group at ZT6. Data are shown as mean ± SEM and data for each ZT are statistically compared using two-way ANOVAs (details are shown in Table S1), followed by Bonferroni post-tests, comparing control vs. TSR and control vs. recovery for each genotype. * p<0.05 in post-test. Post-tests comparing genotypes for each condition (control, TSR and recovery) are shown in Suppl. table ST1. K) Schematic overview of WAT glycolysis and lipogenesis pathways. Transcripts which were found to be changed by TSR are highlighted in red. Sample sizes were 3–4 per group.

We confirmed the up regulation of lipid and carbohydrate metabolism-associated transcripts at ZT18 using qPCR analysis (Fig. 4A–J, Table S1). The activation of key genes involved in glucose uptake (*Slc2a4*) or glycolysis (e.g. *Hk2*, *Pfkfb3*) as well as genes involved in lipogenesis (e.g. *Acaca, Dgat2*) suggests a shift from glucose to lipid storage (Fig. 4K). We also tested the expression levels of these transcripts during recovery from TSR. In line with what we had observed for activity and food intake, TSR-induced transcriptional changes were still detectable in various transcripts during recovery (Fig. 4 A–J, Table S1). Expression of rate-limiting glycolytic (*Gapdh* Fig. 4D) as well as a fatty acid biosynthesis enzymes (*Acaca*, Fig. 4F) remained significantly up regulated during recovery, indicating a prolonged disruption of WAT metabolism-associated transcript regulation for at least as long as one week after the end of TSR. We next tested whether clock-deficient *Per1/2* mutants would be protected from TSR-induced transcriptional changes in WAT. In line with what we had observed for food intake, body weight regulation and leptin secretion we found that at ZT18 none of the above-mentioned transcripts was significantly affected by TSR in *Per1/2* mutants (Fig. 4A–J). Importantly, baseline differences were only detected for *Mogat2* expression at ZT18 (Table S1). A two-factor analysis revealed that many of the genes showed a significant interaction effect (Table S1), suggesting a differential effect of TSR between wild-types and mutants. Given that many of the transcripts we found changed by TSR are clock-controlled, the possibility exists that TSR induced a phase shift rather than an induction of glycolytic and lipogenic genes. To test this we analyzed the same transcripts 12 hours earlier at ZT6. At this time point, glycolytic and lipogenic transcripts were either not changed or induced (*Gapdh*, *Mogat2*, *Dgat2*, *Agpat2*) during TSR or recovery in wild-type animals, indicating that TSR did not simply cause a phase-shift in clock-regulated metabolism-associated genes (Fig. 4 A–J). Similar to what we had observed at ZT18, TSR-induced transcriptional changes were absent in *Per1/2* mutants at ZT6 with the exception of *Pfkfb3*, which was acutely down-regulated by TSR (Fig. 4C, Table S1). In summary, WAT transcriptional changes suggest an increased lipogenic potential in wild-type mice, an effect that is not seen in *Per1/2* mutants.

To test whether the TSR-induced transcriptional changes were accompanied by alterations of blood metabolites associated with energy homeostasis, we measured plasma levels of glucose, non-esterified fatty acids (NEFAs), glycerol, and triglycerides. Levels of all measured metabolites were significantly different between genotypes (Table 1, Table S1), and glucose, glycerol and triglyceride levels were differentially regulated by TSR in wild-types and *Per1/2* mutant animals (significant interaction effect, Table S1). In addition, post tests revealed significant effects of TSR on glucose and triglyceride levels at ZT6 in wild-types (Table 1) as well as genotype differences during control, TSR and recovery conditions at ZT6 and ZT18.

**Table 1:** Effects of TSR on plasma metabolite levels in wild-types and *Per1/2* mutants.

	Wild-type ZT6			*Per1/2* ZT6		
Metabolite	Con	TSR	Rec	Con	TSR	Rec
Glucose (mg/dl)	156±14	210±14*	181±2	258±10#	192±3*	221.18
NEFAs (mM)	1.13±0.10	1.41±0.19	1.43±0.16	1.13±0.14	0.78±0.07#	0.95±0.04
Glycerol (mM)	0.56±0.07	0.68±0.03	0.94±016	0.69±0.07	0.47±0.04	0.44±0.03#
Triglycerides (mM)	0.62±0.11	0.96±0.06*	0.76±0.06	0.57±0.07	0.49±0.05#	0.54±0.05

	Wild-type ZT18			*Per1/2* ZT18		
Metabolite	Con	TSR	Rec	Con	TSR	Rec
Glucose (mg/dl)	168±7	170±5	174±5	206±5#	187±14	210±11#
NEFAs (mM)	0.95±0.13	1.19±0.21	0.92±0.12	1.23±0.08	0.9±0.23	1.27±0.23
Glycerol (mM)	0.44±0.07	0.72±0.11	0.64±0.07	0.85±0.15#	0.85±0.13	0.66±0.06
Triglycerides (mM)	0.67±0.09	0.82±0.1	0.79±0.12	0.76±0.03	0.75±0.09	0.86±0.03

Plasma metabolites were measured at ZT6 and ZT18 in control conditions, on the last day of TSR and on the 7^th^ day of recovery for wild-type and *Per1/2* mutant animals. Data are shown as mean +/− SEM. Sample sizes were 3–5 per group.

* : p<0.05 compared to control conditions in the same genotype,

# : p<0.05 compared to wild-type in the same condition using 2-way ANOVA and Bonferroni post-tests. Statistical details are shown in Table S1. NEFAs: non-esterified fatty acids.

## Discussion

We show that five days of timed sleep restriction (TSR) resulted in alterations of food intake, body weight homeostasis and plasma leptin, glucose, and triglyceride levels in wild-type mice. In addition we found significant transcriptional reprogramming of WAT indicative of increased lipogenesis. Interestingly, some of these effects were sustained for at least one week after the end of the intervention. The lack of a functional clock in *Per1/2* double mutants prevented most of the non-behavioral effects of TSR, suggesting a role for the circadian clock in regulating the metabolic effects of sleep disruption.

### TSR alters activity profiles, food intake, leptin, blood metabolites, and bodyweight

Our gentle handling sleep restriction procedure did not increase total activity nor did we observe major changes in plasma corticosterone levels, suggesting a relatively stress-free procedure. Diurnal activity patterns were changed with a decrease in activity in the second half of the normal active phase. Interestingly changes in activity were still sustained one week after the end of the intervention, reminiscent of rotating shift workers, who report disrupted sleep/wake cycles even after the end of night shift intervals [43].

We observed TSR-induced hyperphagy, which was also sustained during recovery. In a more chronic situation such long-term changes could lead to increased body mass, as has been reported in shift workers and in people with short sleep times [7], [10], [52], but also in mouse models of circadian disruption [41], [53]. In our model, body weight was not increased during the time of TSR despite increased food intake, which is in accordance with previous work in rodents using different sleep restriction procedures [44], [45], [46]. It has been shown that this state of negative energy balance during sleep restriction in rodents reflects increased metabolic rate due to brown adipose tissue-mediated thermogenesis [54]. During the recovery week we observed a clear tendency towards increased body weight gain, which is in line with what has been observed in human shift workers [55]. Thus by measuring not only the acute but also more long-term effects of TSR, we obtain a body weight phenotype in mice, which resembles more a human shift work situation.

Despite increased food intake, plasma leptin levels were elevated by TSR. The literature on the correlation between sleep loss and leptin regulation in humans is controversial, with some studies showing increased leptin with reduced sleep [47], [56], some showing changes in circadian variation of leptin [48], [57], whereas others showing a reduction in leptin upon sleep restriction [52], [58]. Previous rodent studies have mostly reported reduced leptin levels upon sleep restriction [22], [23], [24]. This discrepancy between studies is most likely attributable to either the method and protocol of sleep restriction or the circadian timing of plasma collections. Studies in rodents are particularly difficult to compare, because the methods of sleep restriction range from total sleep deprivation to specific REM sleep deprivation to partial sleep deprivation. Moreover, these methods are likely to differ especially with respect to the amount of stress induction, but also with respect to their effect on activity and, thus, energy expenditure. Our gentle handling protocol resulted in largely unaffected corticosterone and total activity levels. The hyperleptinemia we observe in the absence of a reduction of food intake is a potential indicator of the development of leptin resistance, a common feature of obesity and an early marker of metabolic syndrome [59], [60].

In addition, blood metabolites (glucose, triglycerides) were changed by TSR in wild-types. However, overall the effects of TSR on blood metabolite levels were moderate and – with the exception of glycerol – were no longer significantly different from control levels after one week of recovery. This is likely due to the relatively short-term sleep disruption protocol that we used. The fact that blood glucose and triglyceride levels were affected suggests an involvement of the liver. In line with this we had previously shown that two weeks of TSR cause profound changes in hepatic transcriptome regulation and glucose metabolism [22]. This also suggests that more chronic interventions would potentially lead to more pronounced and longer-lasting effects on blood metabolites.

### TSR-induced transcriptional reprogramming of WAT in wild-type mice

TSR-induced changes in circulating leptin levels suggested physiological alterations in WAT. We observed similar changes in the expression levels of *leptin* mRNA in WAT, which prompted us to analyze the underlying transcriptome changes by microarray analysis. Gene ontology analysis of regulated transcripts revealed an over-representation of genes encoding for key enzymes of glycolytic and lipogenic pathways. *Glut4* (*Slc2a4*), encoding for the insulin-stimulated glucose transporter was up regulated as was *Hk2*, the protein product of which (Hexokinase 2) regulates the first and non-reversible step of glycolysis. In addition, *Pfkfb3* expression was increased. PFKFB3 regulates glycolysis by controlling the levels of fructose-2,6-bisphosphate (F-2,6-BP), which in turn regulates the enzymatic activity of Phosphofructokinase-1. Moreover, transcript levels of *Gapdh*, encoding the other rate-limiting enzyme of glycolysis, were increased by TSR. These changes strongly suggest increased glycolytic potential. The main fate of glycolytic products in WAT is the lipogenic pathway. In line with this, we found TSR-induced transcriptional changes in many important genes involved in lipid biosynthesis. *Acetyl-CoA carboxylase* (*Acaca*), the protein product of which catalyzes the irreversible carboxylation of acetyl-coA to malonyl-CoA was more than 4-fold up regulated at ZT18. In addition major regulators of triacyl-glyceride synthesis such as *Agpat*, *Mogat* and *Dgat* were up regulated by TSR, indicating that not only *de novo* fatty acid synthesis appears increased, but also triglyceride production and storage in adipocytes seems up regulated. The activation of *Gpd1*, which represents a major link between glucose and lipid metabolism is in accordance with our idea of a shift from glucose to fat storage. In a more long-term perspective transcriptional up regulation in lipogenic pathways in adipose tissue may promote increased body mass and adiposity, which has been observed in shift workers as well as in short sleepers [5], [10]. Transcriptional changes in WAT were most pronounced at ZT18, however, some genes were also up-regulated at ZT6. None of the genes was significantly down-regulated at ZT6 in wild-type WAT, making it unlikely that the main effect of TSR was a phase-shift of WAT transcription. However, in order to accurately interpret the phase effects of TSR on WAT transcripts more time points would have to be analyzed. Interestingly, there is evidence that many of the genes, which were changed by TSR, are under the control of the circadian clock: *Pfkfb3*, *Hk2*, *Dgat2* are rhythmically expressed in adipose tissues [61] and *Gpd1*, *Dgat2*, and *Acaca* are rhythmically expressed in liver [62]. Moreover *Gpd1*, *Dgat2*, *Pfkfb3* and *Slc2a4* are differentially expressed in the liver of clock mutant mice [62] and binding of BMAL1 to their promoters was shown [63]. Although expression and promoter binding data from other tissues should be interpreted with caution, this suggests that a number of the TSR targets we have identified may at the same time be regulated by the circadian clock, strengthening the link between the clock, sleep and metabolism. In line with this many canonical clock genes were also regulated by TSR as shown before for other tissues [22], [64].

### Prolonged effects of TSR on adipose transcription

Interestingly, some of the transcriptional changes, which we observed during TSR were not yet fully restored one week after the end of TSR. It is remarkable that even a relatively short time of sleep disruption is sufficient to induce molecular changes lasting for at least one week after the end of the actual intervention. Along that line it has been shown that the effects of shift work are not fully reversible after people stop working shifts and metabolic syndrome is more prevalent among former shift workers [65]. Of course it is very interesting to speculate about the mechanism of such prolonged effects on transcriptional regulation. A recent report suggests a contribution of epigenetic mechanisms: long term shift work was shown to have profound effects on wide-spread alterations in methylation states of genes [66]. Of special interest for our study is that the authors also found circadian clock genes to be differentially methylated by long-term shift work. Accumulating evidence suggests that epigenetic mechanisms are important in regulating the circadian clock and that, vice versa, the circadian clock rhythmically regulates chromatin remodeling (reviewed in [67]). Moreover the important circadian protein CLOCK has histone acetyl-transferase activity [68]. In addition the epigenetic regulation of metabolism is a well-established phenomenon [69], [70]. Thus it is intriguing to suggest that the prolonged transcriptional changes we observe after TSR may result from altered epigenetic regulation. In such a scenario modifiers of chromatin-remodeling enzymes would offer new potential therapeutic targets or even allow the development of preventative medicine against the metabolic effects of sleep disruption.

### In *Per1/2* mutants TSR effects are blunted

We predicted that the absence of a circadian clock in *Per1/2* mutants should protect these animals from at least some of the non-behavioral effects of TSR. Indeed we found no TSR-mediated change in food intake and plasma metabolite and leptin levels in these mice – with the exception of blood glucose which was regulated in the opposite direction as in wild-types. Of note, glucose, glycerol, leptin levels were already elevated at baseline conditions in *Per1/2* mutants compared to wild-types, which is in line with what has been published earlier [71]. Elevated leptin together with unchanged baseline levels of food intake might be an indicator of an innate state of leptin resistance in *Per1/2* mutants. One might argue that in the mutants such disturbed metabolic baseline state may prevent any further deterioration during TSR. On the other hand, blood glucose was still affected and much stronger effects on leptin secretion have been shown after diet manipulation [49]. In addition, transcripts in lipogenic and glycolytic pathways, which we found to be regulated by TSR in wild-type mice, were largely un-affected in *Per1/2* mutants. This indicates that in contrast to wild-type mice, lipogenesis may not be increased by TSR in *Per1/2* mutants, which in turn would protect them from increased fat storage and body weight gain. In line with this, *Per1/2* mutants showed decreased bodyweight gain during recovery. Nevertheless, TSR had strong behavioral effects on *Per1/2* mutants, as their activity pattern changed more dramatically than that of wild-types. *Per1/2* mutants completely lost their normal synchronization relative to the LD cycle, but instead adapted their activity profile to the TSR cycle, becoming predominantly day active, which is in line with an earlier finding showing that clock mutants react differently to non-photic stimuli [72]. This strong effect on activity rhythms, however, did not represent entrainment, but appeared to be an acute response to the TSR procedure as, unlike wild-type mice, *Per1/2* mutants became completely normal again immediately after the end of TSR. In conclusion, the severely blunted transcriptional response of *Per1/2* mutants to TSR suggests that a functional circadian clock may mediate the metabolic response to sleep disruption. We suggest a scenario in which sleep disruption results in clock perturbation which in turn mediates metabolic dysfunction. This chain of effect is broken in the absence of a functional clock as is the case in *Per1/2* mutants. In line with this many of the identified TSR target genes have previously been shown to be under the control of the circadian clock [61], [62], [63]. Alternatively, *Period* genes may have a pleiotropic function in the regulation of adipose physiology. Other clock gene mutants should be analyzed using the same paradigm to clarify this point. In would further be interesting to investigate TSR effects in adipose-specific circadian clock mutants to distinguish between local and systemic circadian regulation in this process.

In summary we show changes in energy homeostasis and WAT transcriptional reprogramming in response to a single five-day shift work protocol in mice. Some aspects of this response are retained for as long as one week after the end of treatment, reminiscent of the long-term metabolic dysregulation observed in human poor sleepers and shift workers. Our finding that the circadian clock gene machinery appears to be involved in this process indicates potential novel therapeutic targets to minimize the adverse metabolic consequences of shift work and sleep disruption.

## Supporting Information

Figure S1 **Interventions necessary to keep animals awake during TSR.** Number of interventions per hour that were necessary to keep wild-type and *Per1/2* mutant mice awake in the first and last hour of SR. Wild-type and *Per1/2* mutant data are pooled. Sample sizes were 10–12 per group and time point. *: p<0.0001, t-test.
(PDF)

Figure S2 **No changes in diurnal corticosterone profiles during or after TSR.** Wild-type diurnal plasma corticosterone profiles under control conditions (black), on the last day of TSR (red) and on the 7^th^ day of recovery (grey). 2-way ANOVA, factor treatment: p = 0.24, factor time: p<0.0001. Sample sizes were 3–4 per group and time point.
(PDF)

Figure S3 **Activity analysis during TSR and recovery in wild-type mice.** A–C: Relative activity levels (relative to control) were analyzed for ZT0–6 (A), ZT6–12 (B) and ZT12–18 (C) using a one-way ANOVA and Bonferroni post-tests comparing control vs. TSR and control vs. recovery. *: p<0.05 in post-test. D) Total activity levels are compared between control, TSR and recovery. No significant differences between conditions were detectable using a one-way ANOVA.
(PDF)

Figure S4 **Leptin mRNA levels are up regulated by TSR.** Expression of leptin in epididymal WAT at ZT18 in control conditions and on the last day of TSR. Data are shown as mean ± SEM. * p<0.05, t-test. Sample sizes were 3–4 per group.
(PDF)

Table S1 **Two-way ANOVA analysis comparing all measured parameters between wild-types and** ***Per1/2*** **mutants.** Two-way ANOVA main effects are described with p-values (p), F-values (F) and degrees of freedom (df) for each effect and residual degrees of freedom (Res. df). For each post-test p-values are given. Post-tests were used to compare effects between control and TSR and control and recovery conditions for each genotype. In addition, cross-genotype comparisons were performed comparing wild-types with *Per1/2* mutants during control, TSR and recovery. Transcription and blood metabolites were analyzed separately for ZT6 and ZT18.
(PDF)

Table S2 **Genes, which were at least 2-fold up or down regulated by TSR at ZT18.** Genes are sorted for fold-change; p-values were obtained using Student's t-tests with Benjamini-Hochberg correction for multiple testing.
(PDF)
