# Supplementary figures and images for: Circadian Clock Genes Per1 and Per2 Regulate the Response of Metabolism-Associated Transcripts to Sleep Disruption
Source: PLoS One. 2012 Dec 28;7(12):e52983. doi: 10.1371/journal.pone.0052983 (PMC3532432; doi:10.1371/journal.pone.0052983)

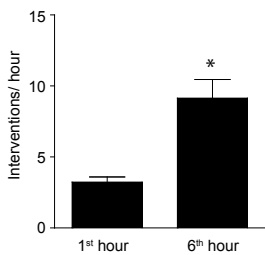

Supplement: Figure S1 — Interventions necessary to keep animals awake during TSR. Number of interventions per hour that were necessary to keep wild-type and Per1/2 mutant mice awake in the first and last hour of SR. Wild-type and Per1/2 mutant data are pooled. Sample sizes were 10–12 per group and time point. *: p<0.0001, t-test. (PDF) [file pone.0052983.s001.pdf]

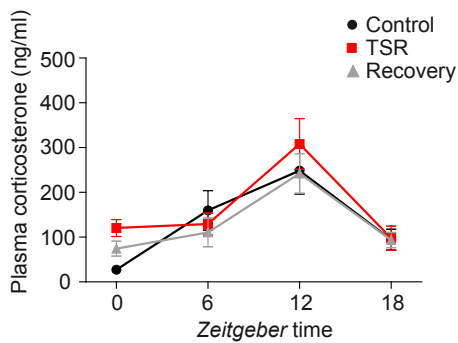

Supplement: Figure S2 — No changes in diurnal corticosterone profiles during or after TSR. Wild-type diurnal plasma corticosterone profiles under control conditions (black), on the last day of TSR (red) and on the 7th day of recovery (grey). 2-way ANOVA, factor treatment: p = 0.24, factor time: p<0.0001. Sample sizes were 3–4 per group and time point. (PDF) [file pone.0052983.s002.pdf]

**A**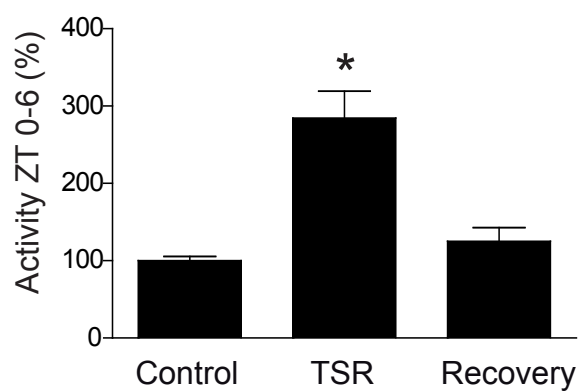**B**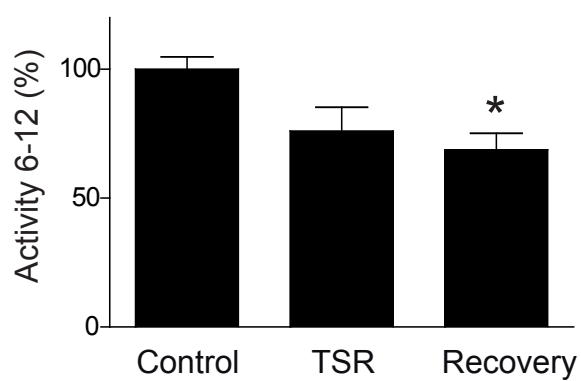**C**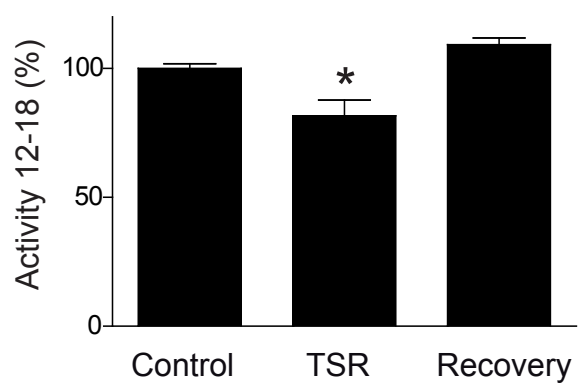**D**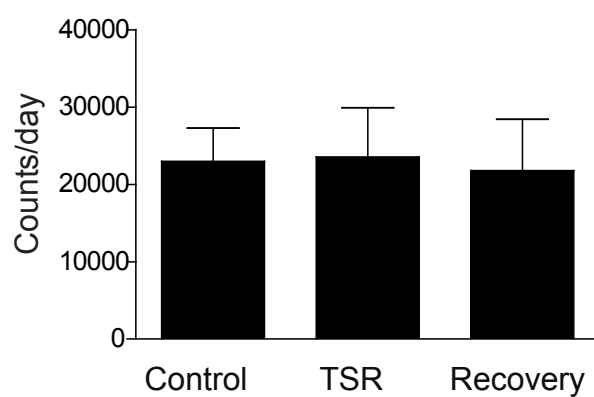

Supplement: Figure S3 — Activity analysis during TSR and recovery in wild-type mice. A–C: Relative activity levels (relative to control) were analyzed for ZT0–6 (A), ZT6–12 (B) and ZT12–18 (C) using a one-way ANOVA and Bonferroni post-tests comparing control vs. TSR and control vs. recovery. *: p<0.05 in post-test. D) Total activity levels are compared between control, TSR and recovery. No significant differences between conditions were detectable using a one-way ANOVA. (PDF) [file pone.0052983.s003.pdf]

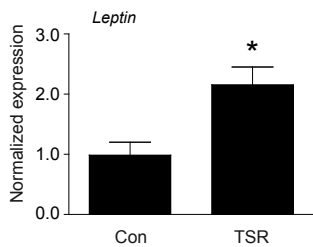

Supplement: Figure S4 — Leptin mRNA levels are up regulated by TSR. Expression of leptin in epididymal WAT at ZT18 in control conditions and on the last day of TSR. Data are shown as mean ± SEM. * p<0.05, t-test. Sample sizes were 3–4 per group. (PDF) [file pone.0052983.s004.pdf]
