# Supplementary material for: Circadian Clock Genes Per1 and Per2 Regulate the Response of Metabolism-Associated Transcripts to Sleep Disruption
Source: PLoS One. 2012 Dec 28;7(12):e52983. doi: 10.1371/journal.pone.0052983 (PMC3532432; doi:10.1371/journal.pone.0052983)
Supplement: Table S1 — Two-way ANOVA analysis comparing all measured parameters between wild-types and Per1/2 mutants. Two-way ANOVA main effects are described with p-values (p), F-values (F) and degrees of freedom (df) for each effect and residual degrees of freedom (Res. df). For each post-test p-values are given. Post-tests were used to compare effects between control and TSR and control and recovery conditions for each genotype. In addition, cross-genotype comparisons were performed comparing wild-types with Per1/2 mutants during control, TSR and recovery. Transcription and blood metabolites were analyzed separately for ZT6 and ZT18. (PDF) [file pone.0052983.s005.pdf]

	2-way-ANOVA main effects				Posthoc tests effect sleep				Posthoc tests effect genotype		
					Wild-type		*Per1/2*		Wildtype vs. *Per1/2*		
	Sleep (p;F;df)	Genotype (p;F;df)	Interaction (p;F;df)	Res. df	Con vs TSR	Con vs Rec	Con vs TSR	Con vs Rec	Control	TSR	Rec
**Bodyweight**	< 0.0001; 65.84; 2	0.2119; 1.571; 1	0.0499; 3.057; 2	157	< 0.001	> 0.05	< 0.001	< 0.05	> 0.05	> 0.05	> 0.05
**Food intake**	0.0136; 4.979; 2	0.0051; 9.146; 1	0.4667; 0.7818; 2	30	< 0.05	> 0.05	> 0.05	> 0.05	> 0.05	< 0.05	> 0.05
**Leptin**	0.0392; 3.794; 2	0.0165; 6.790; 1	0.3756; 1.026; 2	21	< 0.05	> 0.05	> 0.05	> 0.05	< 0.05	> 0.05	> 0.05
**Activity ZT18-24**	< 0.0001; 66.76; 2	0.0010; 14.28; 1	0.0153; 5.038; 2	23	< 0.001	< 0.01	< 0.001	> 0.05	> 0.05	< 0.001	> 0.05
**Transcription**											
**Slc2a4 ZT6**	0.4816; 0.7630; 2	0.001; 15.67; 1	0.5672; 0.5864; 2	17	> 0.05	> 0.05	> 0.05	> 0.05	> 0.05	> 0.05	< 0.05
**Slc2a4 ZT18**	0.0552; 3.417; 2	0.0282; 5.696; 1	0.0202; 4.882; 2	18	< 0.05	> 0.05	> 0.05	> 0.05	> 0.05	< 0.01	> 0.05
**HK2 ZT6**	0.684; 0.3884; 2	0.4886; 0.5010; 1	0.2867; 1.346; 2	17	> 0.05	> 0.05	> 0.05	> 0.05	> 0.05	> 0.05	> 0.05
**HK2 ZT18**	0.0588; 3.331; 2	0.0014; 14.12; 1	0.0843; 2.847; 2	18	< 0.01	< 0.05	> 0.05	> 0.05	> 0.05	< 0.05	< 0.05
**Pfkfb3 ZT6**	0.5206; 0.6784; 2	0.9147; 0.01183; 1	0.0386; 3.966; 2	17	> 0.05	> 0.05	< 0.05	> 0.05	> 0.05	> 0.05	> 0.05
**Pfkfb3 ZT18**	0.1295; 2.294; 2	0.8091; 0.06012; 1	0.0346; 4.080; 2	18	< 0.01	> 0.05	> 0.05	> 0.05	> 0.05	> 0.05	> 0.05
**Gapdh ZT6**	0.0205; 4.929; 2	0.0163; 7.104; 1	0.076; 3.010; 2	17	> 0.05	< 0.01	> 0.05	> 0.05	> 0.05	> 0.05	< 0.01
**Gapdh ZT18**	0.1079; 2.526; 2	0.0002; 22.65; 1	0.0465; 3.656; 2	18	< 0.05	< 0.05	> 0.05	> 0.05	> 0.05	< 0.01	< 0.01
**Gpd1 ZT6**	0.4176; 0.9198; 2	0.0286; 5.723; 1	0.8889; 0.1186; 2	17	> 0.05	> 0.05	> 0.05	> 0.05	> 0.05	> 0.05	> 0.05
**Gpd1 ZT18**	0.0807; 2.904; 2	0.4624; 0.5638; 1	0.3875; 0.9997; 2	18	< 0.05	> 0.05	> 0.05	> 0.05	> 0.05	> 0.05	> 0.05
**Acaca ZT6**	0.4324; 0.8811; 2	0.0043; 10.85; 1	0.4997; 0.7228; 2	17	> 0.05	> 0.05	> 0.05	> 0.05	> 0.05	> 0.05	> 0.05
**Acaca ZT18**	0.0894; 2.770; 2	0.0003; 19.81; 1	0.0597; 3.309; 2	18	< 0.05	< 0.05	> 0.05	> 0.05	> 0.05	< 0.01	< 0.01
**Mogat2 ZT6**	0.0133; 5.627; 2	0.1171; 0.1171; 1	0.0432; 3.802; 2	17	> 0.05	< 0.01	> 0.05	> 0.05	> 0.05	> 0.05	< 0.05
**Mogat2 ZT18**	0.216; 1.671; 2	0.106; 2.896; 1	0.0118; 5.739; 2	18	> 0.05	< 0.01	> 0.05	> 0.05	< 0.01	> 0.05	> 0.05
**Dgat2 ZT6**	0.0538; 3.487; 2	0.878; 0.02429; 1	0.0765; 3.001; 2	17	> 0.05	< 0.01	> 0.05	> 0.05	> 0.05	> 0.05	> 0.05
**Dgat2 ZT18**	0.5303; 0.6573; 2	0.4382; 0.6285; 1	0.0156; 5.284; 2	18	< 0.05	> 0.05	> 0.05	> 0.05	> 0.05	> 0.05	> 0.05
**Agpat1 ZT6**	0.228; 1.615; 2	0.239; 1.489; 1	0.1037; 2.596; 2	17	> 0.05	> 0.05	> 0.05	> 0.05	> 0.05	> 0.05	> 0.05
**Agpat1 ZT18**	0.3975; 0.9745; 2	0.004; 11.08; 1	0.0931; 2.738; 2	18	< 0.05	> 0.05	> 0.05	> 0.05	> 0.05	< 0.01	> 0.05
**Agpat2 ZT6**	0.0220; 4.816; 2	0.0061; 9.796; 1	0.0455; 3.726; 2	17	> 0.05	< 0.01	> 0.05	> 0.05	> 0.05	> 0.05	< 0.01
**Agpat2 ZT18**	0.0714; 3.067; 2	< 0.0001; 30.48; 1	0.058; 3.350; 2	18	< 0.01	< 0.05	> 0.05	> 0.05	> 0.05	< 0.01	< 0.01
**Blood metabolites**											
**Glucose ZT6**	0.7946; 0.2334; 2	0.0003; 22.59; 1	0.0001; 17.99; 2	15	< 0.01	> 0.05	< 0.001	> 0.05	< 0.001	> 0.05	> 0.05
**Glucose ZT18**	0.1436; 2.142; 2	< 0.0001; 24; 1	0.2882; 1.325; 2	20	> 0.05	> 0.05	> 0.05	> 0.05	< 0.05	> 0.05	< 0.01
**NEFAs ZT6**	0.7227; 0.3314; 2	0.0022; 13.26; 1	0.0603; 3.366; 2	16	> 0.05	> 0.05	> 0.05	> 0.05	> 0.05	< 0.01	< 0.05
**NEFAs ZT18**	0.9587; 0.04226; 2	0.4626; 0.5648; 1	0.1807; 1.895; 2	17	> 0.05	> 0.05	> 0.05	> 0.05	> 0.05	> 0.05	> 0.05
**Glycerol ZT6**	0.3641; 1.073; 2	0.0109; 8.168; 1	0.0052; 7.274; 2	17	> 0.05	< 0.05	> 0.05	> 0.05	> 0.05	> 0.05	< 0.01
**Glycerol ZT18**	0.3984; 0.9689; 2	0.0302; 5.535; 1	0.1246; 2.343; 2	18	> 0.05	> 0.05	> 0.05	> 0.05	< 0.05	> 0.05	> 0.05
**Triglycerides ZT6**	0.1858; 1.873; 2	0.0003; 20.86; 1	0.0173; 5.283; 2	16	< 0.01	> 0.05	> 0.05	> 0.05	> 0.05	< 0.001	> 0.05
**Triglycerides ZT18**	0.4498; 0.1915; 2	0.6669; 0.8355; 1	0.5638; 0.5918; 2	18	> 0.05	> 0.05	> 0.05	> 0.05	> 0.05	> 0.05	> 0.05
