# Supplementary material for: Circadian Clock Genes Per1 and Per2 Regulate the Response of Metabolism-Associated Transcripts to Sleep Disruption
Source: PLoS One. 2012 Dec 28;7(12):e52983. doi: 10.1371/journal.pone.0052983 (PMC3532432; doi:10.1371/journal.pone.0052983)
Supplement: Table S2 — Genes, which were at least 2-fold up or down regulated by TSR at ZT18. Genes are sorted for fold-change; p-values were obtained using Student's t-tests with Benjamini-Hochberg correction for multiple testing. (PDF) [file pone.0052983.s006.pdf]

Probe Set ID	Entrez Gene ID	Offical Gene Symbol	P-value	Fold change
1419622_at	22238	Ugt2b5	0.022780	116.68
1428079_at	110135	Fgb	0.015765	102.80
1451580_a_at	22139	Ttr	0.000427	102.68
1419233_x_at	11806	Apoa1	0.003636	98.14
1424973_at	56388	Cyp3a25	0.035228	96.88
1455201_x_at	11806	Apoa1	0.010759	87.28
1417422_at	14711	Gnmt	0.041443	86.51
1448680_at	20702	Serpina1c	0.016193	44.17
1454608_x_at	22139	Ttr	0.009673	39.03
1450624_at	12116	Bhmt	0.003518	38.74
1455093_a_at	11625	Ahsg	0.012685	36.14
1455913_x_at	22139	Ttr	0.008319	35.40
1451513_x_at	20700 /// 20701	Serpina1a /// Serpina1b	0.009286	34.26
1416649_at	11699	Ambp	0.025015	32.61
1448764_a_at	14080	Fabp1	0.022127	32.22
1417246_at	11287	Pzp	0.003410	31.11
1418239_at	103161	Apof	0.043546	29.57
1427422_at	624219	Gm6484	0.018019	29.34
1418113_at	13101	Cyp2d10	0.020175	28.90
1426547_at	14473	Gc	0.020848	28.62
1427631_x_at	17842	Mup3	0.035734	27.35
1418771_a_at	56373	Cpb2	0.020531	27.11
1438840_x_at	11806	Apoa1	0.008745	24.96
1419232_a_at	11806	Apoa1	0.002041	24.93
1448723_at	54150	Rdh7	0.003523	24.90
1425260_at	11657	Alb	0.010882	24.81
1417556_at	14080	Fabp1	0.007482	24.71
1416025_at	99571	Fgg	0.010008	21.01
1418282_x_at	20701	Serpina1b	0.011968	18.81
1449280_at	71690	Esm1	0.003483	17.82
1417085_at	83702	Akr1c6	0.007638	17.47
1417835_at	17836	Mug1	0.003538	17.07
1434927_at	29818	Hspb7	0.007793	16.67
1448852_at	19733	Rgn	0.011636	16.45
1449321_x_at	20700 /// 20701 /// 20702 /// 20703 /// 20704	Serpina1a /// Serpina1b /// Serpina1c /// Serpina1d /// Serpina1e	0.006637	15.35
1417950_a_at	11807	Apoa2	0.014308	15.14
1448854_s_at	17835 /// 17836 /// 17837	Mug-ps1 /// Mug1 /// Mug2	0.003359	14.60
1416913_at	13884	Ces1c	0.031146	14.25
1421037_at	18143	Npas2	0.013273	13.90
1455540_at	227231	Cps1	0.023960	13.72
1416676_at	16644	Kng1	0.047162	13.68
1450715_at	13077	Cyp1a2	0.008336	13.46

1420553_x_at	20700	Serpina1a	0.021777	13.09
1424279_at	14161	Fga	0.012762	13.04
1448889_at	69354	Slc38a4	0.046609	12.60
1452986_at	15233	Hgd	0.013385	12.22
1419131_at	14060	F13b	0.008729	12.13
1423147_at	11720	Mat1a	0.011764	12.05
1451194_at	230163	Aldob	0.001905	11.94
1423944_at	15458	Hpx	0.013183	11.74
1424898_at	20493	Slc10a1	0.042932	11.70
1420525_a_at	18416	Otc	0.020440	10.68
1416809_at	13112	Cyp3a11	0.020270	10.54
1420420_at	15112	Hao1	0.017694	10.04
1422815_at	12279	C9	0.015308	10.00
1418897_at	14061	F2	0.007665	9.94
1421290_at	29818	Hspb7	0.008308	9.46
1424934_at	71773	Ugt2b1	0.013200	9.39
1416729_at	18815	Plg	0.035320	9.13
1448470_at	14121	Fbp1	0.029619	9.04
1448813_at	67758	Aadac	0.025483	8.79
1425326_at	104112	Acly	0.015180	8.68
1451600_s_at	13909 /// 382053	Ces3a /// Ces3b	0.019076	8.67
1418914_s_at	64918	Bhmt2	0.038658	8.56
1455593_at	238055	Apob	0.016243	8.13
1420465_s_at	100039008 /// 100039054 /// 100039089 /// 100039150 /// 100041658 /// 100041687 /// 100048885 /// 100189605 /// 17840 /// 17841	LOC100048885 /// Mup1 /// Mup10 /// Mup12 /// Mup13 /// Mup15 /// Mup19 /// Mup2 /// Mup7 /// Mup8	0.016168	8.06
1426154_s_at	100039008 /// 100048884 /// 17840 /// 17841 /// 17842	LOC100048884 /// Mup1 /// Mup10 /// Mup2 /// Mup3	0.027618	7.54
1427393_at	14071	F9	0.042861	7.43
1420357_s_at	22445 /// 22446 /// 574437 /// 630164	LOC630164 /// Xlr3a /// Xlr3b /// Xlr3c	0.017152	7.39
1452494_s_at	28253	Slco1b2	0.011554	7.38
1449112_at	26459	Slc27a5	0.036694	7.27
1421946_at	12944	Crp	0.008355	7.24
1417909_at	11905	Serpinc1	0.030896	7.14
1418278_at	11814	Apoc3	0.012390	6.86
1419549_at	11846	Arg1	0.014815	6.78
1455490_at	18703	Pigr	0.040788	6.64
1424618_at	15445	Hpd	0.003526	6.64
1418025_at	20893	Bhlhe40	0.003584	6.64

1422604_at	22262	Uox	0.003366	5.96
1416639_at	56485	Slc2a5	0.007809	5.96
1416677_at	11818	Apoh	0.018706	5.51
1421991_a_at	16010	Igfbp4	0.033531	5.44
1454638_a_at	18478	Pah	0.034035	5.44
1428012_at	230558	C8a	0.027913	5.37
1449519_at	13197	Gadd45a	0.010294	5.33
1417776_at	12007	Azgp1	0.010066	5.23
1418937_at	13371	Dio2	0.032669	5.22
1427455_x_at	110759 /// 16071 /// 16114 /// 384419 /// 546213 /// 637227	Gm10883 /// Gm1420 /// Gm7202 /// Igk-C /// Igk-J1 /// Igk-V28	0.009595	5.07
1417403_at	170439	Elovl6	0.008758	4.98
1415823_at	20250	Scd2	0.032657	4.93
1427042_at	105853	Mal2	0.028446	4.92
1420735_at	14409	Gabrr2	0.029046	4.83
1424273_at	226105	Cyp2c70	0.027223	4.74
1427735_a_at	11459	Acta1	0.006359	4.69
1453839_a_at	74116	Pi16	0.030674	4.61
1418787_at	17195	Mbl2	0.027978	4.48
1419196_at	84506	Hamp	0.038875	4.47
1417404_at	170439	Elovl6	0.003551	4.33
1423978_at	104175	Sbk1	0.015320	4.26
1419349_a_at	13105	Cyp2d9	0.022904	4.24
1449449_at	64292	Ptges	0.006745	4.21
1416936_at	11302	Aatk	0.007463	4.20
1431947_at	16835	Ldlr	0.016464	4.12
1427595_at	107476	Acaca	0.035444	4.09
1418724_at	12630	Cfi	0.003490	4.05
1420742_at	11500	Adam7	0.039390	4.01
1425631_at	53412	Ppp1r3c	0.009290	3.97
1449123_at	16426	Itih3	0.024570	3.96
1424722_at	71775	1300017J02Rik	0.026315	3.90
1416455_a_at	12955	Cryab	0.004402	3.88
1451515_s_at	107146	Glyat	0.021406	3.87
1452417_x_at	110759 /// 16071 /// 16114 /// 384419 /// 546213 /// 637227	Gm10883 /// Gm1420 /// Gm7202 /// Igk-C /// Igk-J1 /// Igk-V28	0.043437	3.86
1416204_at	14555	Gpd1	0.010161	3.84
1448710_at	12767	Cxcr4	0.016287	3.83
1455099_at	233549	Mogat2	0.015078	3.80
1421679_a_at	12575	Cdkn1a	0.012836	3.70
1422571_at	21826	Thbs2	0.032078	3.63
1420722_at	12686	Elovl3	0.003313	3.63
1451190_a_at	104175	Sbk1	0.031201	3.62

1427660_x_at	110759 /// 16071 /// 16114 /// 384419 /// 546213 /// 637227	Gm10883 /// Gm1420 /// Gm7202 /// Igk-C /// Igk-J1 /// Igk-V28	0.026694	3.60
1430483_a_at	71913	Tmem79	0.013382	3.60
1450718_at	23921	Sh2b2	0.003465	3.55
1421915_a_at	20441	St3gal3	0.012810	3.53
1417413_at	16433	Cuzd1	0.012302	3.50
1426867_at	226049	Dmrt2	0.017738	3.38
AFFX-PyruCarbMur/L09192_MB_at	18563	Pcx	0.023749	3.37
1449309_at	13124	Cyp8b1	0.014579	3.37
1423180_at	16500	Kcnb1	0.033347	3.33
1449219_at	60527	Fads3	0.006806	3.31
1421063_s_at	20646 /// 84704	Snrpn /// Snurf	0.012774	3.29
1424815_at	232493	Gys2	0.001929	3.28
1449551_at	17913	Myo1c	0.008270	3.26
1416955_at	27376	Slc25a10	0.003559	3.26
1419314_at	26944	Tinag	0.046294	3.25
1423244_at	433247	Cyp2c68	0.039841	3.19
1422677_at	67800	Dgat2	0.021877	3.19
1440230_at	244152	Tsku	0.014438	3.15
1422922_at	79456	Recql4	0.012768	3.14
1418932_at	18030	Nfil3	0.031930	3.12
1428005_at	66112	Mosc1	0.011248	3.12
1460642_at	22032	Traf4	0.017364	3.11
1422327_s_at	14380 /// 14381	G6pd2 /// G6pdx	0.005274	3.10
1426215_at	13195	Ddc	0.024089	3.08
AFFX-r2-Bs-dap-5_at	---	---	0.002139	3.07
1450661_x_at	18029	Nfic	0.019001	3.07
1423179_at	16500	Kcnb1	0.025760	3.06
1420943_at	22673	Zfp185	0.005489	3.05
1460223_a_at	13829	Epb4.9	0.011336	3.02
1431464_a_at	54128	Pmm2	0.035750	3.01
1417806_at	64082	Popdc2	0.016390	2.99
1424735_at	227731	Slc25a25	0.011025	2.97
1427619_a_at	231147	Sh3tc1	0.018378	2.97
1453111_a_at	68066	Slc25a39	0.010051	2.95
1451956_a_at	18391	Sigmar1	0.005221	2.93
1450097_s_at	14673	Gna12	0.009270	2.93
1422550_a_at	17760	Mtap6	0.043579	2.92
1449374_at	19193	Pipox	0.033850	2.92
1415959_at	20528	Slc2a4	0.008960	2.92
1430999_a_at	56367	Scoc	0.009285	2.92
1426045_at	16644	Kng1	0.023402	2.89
1418879_at	104943	Fam110c	0.003414	2.89
1449450_at	64292	Ptges	0.016489	2.88

1424758_s_at	217847	Serpina10	0.038947	2.87
1428021_at	78038	Mccc2	0.021281	2.86
1452427_s_at	57874	Ptplad1	0.012571	2.86
1425593_at	212999	Tnpo2	0.046090	2.81
1417430_at	12585	Cdr2	0.002887	2.81
1420917_at	56194	Prpf40a	0.016694	2.80
1450261_a_at	20493	Slc10a1	0.023715	2.80
1433491_at	13822	Epb4.1l2	0.014713	2.78
1419022_a_at	100503183 /// 13806 /// 433182	Eno1 /// Gm5506 /// LOC100503183	0.013213	2.78
1418497_at	14168	Fgf13	0.007457	2.77
1425610_s_at	108148	Galnt2	0.003537	2.76
1416840_at	68041	Mid1ip1	0.011225	2.75
1449555_a_at	59083	Fetub	0.024308	2.73
1423968_at	223337	Ugt3a2	0.041412	2.69
1416487_a_at	22601	Yap1	0.007591	2.69
1417014_at	80888	Hspb8	0.025413	2.67
1417610_at	66113	Apoa5	0.033536	2.64
1424214_at	231440	Parm1	0.003737	2.63
1416794_at	56298	Atl2	0.003568	2.63
1418129_at	74754	Dhcr24	0.019769	2.60
1433691_at	53412	Ppp1r3c	0.027902	2.60
1449817_at	27413	Abcb11	0.032698	2.60
1420816_at	22628	Ywhag	0.025844	2.60
1417308_at	18746	Pkm2	0.003427	2.60
1421889_a_at	11804	Aplp2	0.003600	2.59
1418853_at	28194	Apon	0.047160	2.56
1430780_a_at	29858	Pmm1	0.005899	2.56
1421265_a_at	56190	Rbm38	0.042387	2.56
1455905_at	72503	2610507B11Rik	0.006681	2.55
1452015_at	230279	6330416G13Rik	0.008732	2.54
1425911_a_at	14182	Fgfr1	0.003804	2.52
1452361_at	109331	Rnf20	0.010146	2.52
1450506_a_at	68048	Aen	0.024611	2.50
1420346_at	70392	Asb12	0.003983	2.50
1423557_at	15980	Ifngr2	0.005342	2.50
AFFX-DapX-5_at	---	---	0.029037	2.49
1456676_a_at	170768	Pfkfb3	0.037632	2.49
1448132_at	20509	Slc19a1	0.027582	2.49
1422063_a_at	19305	Pex5	0.016296	2.49
1438476_a_at	107932	Chd4	0.024206	2.48
1418701_at	12846	Comt1	0.015871	2.47
1436343_at	107932	Chd4	0.015098	2.47
1425930_a_at	21428	Mlx	0.023892	2.46
1421992_a_at	16010	Igfbp4	0.017117	2.45
1434393_at	17847	Usp34	0.034968	2.45

1424032_at	74096	Hvcn1	0.021795	2.45
1450035_a_at	56194	Prpf40a	0.005524	2.44
1423947_at	73737	1110008P14Rik	0.008499	2.43
1450259_a_at	20850	Stat5a	0.039197	2.41
1416593_at	93692	Glrx	0.011743	2.40
1423418_at	110196	Fdps	0.005596	2.38
1452862_at	68750	Rreb1	0.034371	2.37
1425792_a_at	19885	Rorc	0.046286	2.37
1418572_x_at	27279	Tnfrsf12a	0.025768	2.36
1450944_at	121021	Cspg4	0.003126	2.35
1419093_at	56720	Tdo2	0.045799	2.35
1419583_at	12418	Cbx4	0.011986	2.35
AFFX-r2-Bs-thr-5_s_at	---	---	0.011785	2.35
1428891_at	231440	Parm1	0.013511	2.34
1418123_at	22248	Unc119	0.003593	2.34
1420657_at	22229	Ucp3	0.007490	2.33
1416853_at	26562	Ncdn	0.013151	2.31
1447982_at	73737	1110008P14Rik	0.043555	2.31
1423706_a_at	110208	Pgd	0.003551	2.31
1449252_at	104943	Fam110c	0.009622	2.30
1451666_at	104112	Acly	0.037619	2.30
1423384_s_at	21766	Tex261	0.008829	2.30
1438387_x_at	21976	Top3b	0.031406	2.29
1455844_at	621998	Gm6277	0.031588	2.28
1421842_a_at	100113398 /// 56214	Adat3 /// Scamp4	0.032325	2.28
1452742_at	67095	Trak1	0.032339	2.28
1421024_at	55979	Agpat1	0.013431	2.28
1418337_at	19895	Rpia	0.029018	2.28
1423905_at	52118	Pvr	0.027922	2.27
1435833_at	---	---	0.007586	2.27
1418247_s_at	93686	Rbfox2	0.025637	2.27
1426726_at	52040 /// 667766	Gm8801 /// Ppp1r10	0.014603	2.26
1424611_x_at	227682	Trub2	0.013200	2.26
1421313_s_at	13043	Cttn	0.039316	2.26
1415822_at	20250	Scd2	0.029056	2.25
AFFX-GapdhMur/M32599_5_at	14433	Gapdh	0.014799	2.25
1426315_a_at	230279	6330416G13Rik	0.016054	2.25
1435950_at	15460	Hr	0.013052	2.25
1421075_s_at	13123	Cyp7b1	0.030422	2.23
1448249_at	14555	Gpd1	0.013344	2.23
1431808_a_at	16427	Itih4	0.008935	2.23
1422996_at	171210	Acot2	0.046534	2.23
1448228_at	16948	Lox	0.012377	2.23
1460460_a_at	70231	Gorasp2	0.038708	2.23

1419703_at	53867	Col5a3	0.002762	2.22
1448945_at	67801	Pllp	0.028883	2.21
1426727_s_at	52040 /// 667766	Gm8801 /// Ppp1r10	0.011253	2.21
1423062_at	16009	Igfbp3	0.022001	2.21
1426756_at	108148	Galnt2	0.034519	2.21
1415807_s_at	20382	Srsf2	0.008700	2.21
1423566_a_at	15505	Hsph1	0.034150	2.20
1424638_at	12575	Cdkn1a	0.008477	2.19
1439075_at	70408	Polr3f	0.041471	2.18
1425099_a_at	11865	Arntl	0.013884	2.18
1449433_at	94045	P2rx5	0.019868	2.18
1423866_at	20714	Serpina3k	0.015171	2.17
1416825_at	20648	Snta1	0.030651	2.17
1420867_at	56334	Tmed2	0.015598	2.17
1417236_at	57440	Ehd3	0.009777	2.16
1454875_a_at	19646	Rbbp4	0.039683	2.15
1424747_at	16562	Kif1c	0.010842	2.15
1448607_at	59027	Nampt	0.033815	2.15
1428669_at	107771	Bmyc	0.029870	2.14
1417151_a_at	18217	Ntsr2	0.012454	2.14
1429566_a_at	15258	Hipk2	0.027573	2.14
1416741_at	12831	Col5a1	0.037278	2.14
AFFX-GapdhMur/M32599_M_at	14433	Gapdh	0.038341	2.13
1450799_at	11517	Adcyap1r1	0.031404	2.13
1438627_x_at	110208	Pgd	0.008758	2.13
1425929_a_at	56736	Rnf14	0.029879	2.12
1430980_a_at	13681	Eif4a1	0.007277	2.12
1422612_at	15277	Hk2	0.002765	2.11
1420619_a_at	14797	Aes	0.016191	2.11
1433448_at	229517	Slc25a44	0.003824	2.11
1425991_a_at	235041	Kank2	0.007870	2.11
1450007_at	56398	1500003O03Rik	0.011766	2.11
1430976_a_at	78523	Mrpl9	0.005990	2.10
1420427_a_at	101437	Dhx32	0.008478	2.09
1420138_at	20509	Slc19a1	0.007133	2.09
1435495_at	11539	Adora1	0.020210	2.09
1430307_a_at	17436	Me1	0.008827	2.08
1427764_a_at	21423	Tcf3	0.015175	2.07
1448231_at	14229	Fkbp5	0.015872	2.07
1455061_a_at	52538	Acaa2	0.010062	2.07
1421074_at	13123	Cyp7b1	0.028337	2.07
1416750_at	18391	Sigmar1	0.005303	2.07
1451015_at	21881	Tkt	0.014452	2.06
1450286_at	18162	Npr3	0.016520	2.06
1415958_at	20528	Slc2a4	0.040380	2.06

1416852_a_at	26562	Ncdn	0.010053	2.06
1448851_a_at	13002	Dnajc5	0.038513	2.05
1424906_at	217430	Pqlc3	0.008541	2.05
1424350_s_at	226856	Lpgat1	0.000759	2.05
1421821_at	16835	Ldlr	0.005563	2.05
1460336_at	19017	Ppargc1a	0.010114	2.05
1421324_a_at	11652	Akt2	0.033506	2.05
1426406_at	67956	Setd8	0.006446	2.05
1449645_s_at	12462	Cct3	0.043616	2.05
1434436_at	75746	Morc4	0.003276	2.04
1453367_a_at	76192	Abhd12	0.009288	2.04
1423334_at	67458	Ergic1	0.022879	2.04
1427100_at	70083	Metrn	0.040458	2.04
1423916_s_at	30853	Mlf2	0.013785	2.04
1426258_at	20660	Sorl1	0.022888	2.03
1453623_a_at	19358	Rad23a	0.029353	2.03
1416660_at	13669	Eif3a	0.040116	2.03
1452256_at	68550	1110002N22Rik	0.005945	2.03
1424167_a_at	29858	Pmm1	0.033087	2.03
1449337_at	56720	Tdo2	0.026039	2.02
1450627_at	11732	Ank	0.003595	2.02
1424610_at	227682	Trub2	0.008900	2.02
1449054_a_at	59092	Pcbp4	0.023504	2.02
1459890_s_at	73737	1110008P14Rik	0.021815	2.02
1451737_at	18708	Pik3r1	0.022826	2.02
1427324_at	330189	Tmem120b	0.002253	2.02
1454197_a_at	108673	Ccdc86	0.012362	2.01
1449137_at	18597	Pdha1	0.005517	2.01
1451625_a_at	69379	C8g	0.036672	2.01
1433460_at	104718	Ttc7b	0.023324	2.01
1420612_s_at	19244	Ptp4a2	0.008481	2.01
1420013_s_at	16987	Lss	0.036876	2.01
1452653_at	68267	Slc25a22	0.006676	2.01
1425329_a_at	109754	Cyb5r3	0.012693	2.00
1455873_a_at	228545	Vps18	0.026431	2.00
1456043_at	216825	Usp22	0.028310	2.00
1450792_at	22177	Tyrobp	0.005494	0.50
1420549_at	14468	Gbp1	0.017294	0.50
1420175_at	52440	Tax1bp1	0.038192	0.50
1425809_at	11770	Fabp4	0.003594	0.50
1425311_at	78792	4930432F04Rik	0.015179	0.50
1418509_at	12409	Cbr2	0.006346	0.49
1417910_at	12428	Ccna2	0.033783	0.49
1419721_at	80885	Niacr1	0.003473	0.49
1417185_at	110454	Ly6a	0.007252	0.49
1421855_at	14190	Fgl2	0.044368	0.49

1418131_at	56045	Samhd1	0.025430	0.49
1427202_at	320204	4833442J19Rik	0.007706	0.49
1455214_at	17342	Mitf	0.012336	0.49
1452207_at	17684	Cited2	0.001973	0.49
1417104_at	13732	Emp3	0.009996	0.49
1451289_at	13175	Dclk1	0.021284	0.49
1417399_at	14456	Gas6	0.002803	0.49
1448995_at	56744	Pf4	0.007887	0.49
1422875_at	12523	Cd84	0.037286	0.48
1419605_at	17312	Clec10a	0.033501	0.48
1451264_at	319710	Frmd6	0.014607	0.48
1422789_at	19378	Aldh1a2	0.027302	0.48
1448789_at	56847	Aldh1a3	0.047956	0.48
1421685_at	69810	Clec4b1	0.008267	0.48
1423439_at	18534	Pck1	0.015408	0.48
1439283_at	100273	Osbpl9	0.021205	0.47
1449308_at	12274	C6	0.040370	0.47
1434437_x_at	20135	Rrm2	0.032207	0.47
1422903_at	17084	Ly86	0.032175	0.47
1440865_at	213002	Ifitm6	0.029734	0.47
1416016_at	21354	Tap1	0.005984	0.47
1437726_x_at	12260	C1qb	0.003663	0.47
1418156_at	57814	Kcne4	0.025816	0.47
1429761_at	104001	Rtn1	0.047048	0.46
1419194_s_at	63986	Gmfg	0.016833	0.46
1417141_at	16145	Igtp	0.008486	0.46
1452405_x_at	100038850	A130082M07Rik	0.027280	0.46
1418507_s_at	216233	Socs2	0.007487	0.46
1449630_s_at	226778	Mark1	0.033862	0.46
1449461_at	63954	Rbp7	0.016242	0.46
1418826_at	69774	Ms4a6b	0.028325	0.46
1450783_at	15957	Ifit1	0.028041	0.45
1424208_at	19219	Ptger4	0.004519	0.45
1417346_at	66824	Pycard	0.040124	0.45
1421087_at	18628	Per3	0.003790	0.45
1450488_at	56221	Ccl24	0.007450	0.45
1417961_a_at	20128	Trim30a	0.020351	0.45
1424775_at	246730	Oas1a	0.029446	0.45
1419193_a_at	63986	Gmfg	0.013876	0.44
1419004_s_at	12044 /// 12045 /// 12047	Bcl2a1a /// Bcl2a1b /// Bcl2a1d	0.010235	0.44
1449015_at	57262	Retnla	0.032070	0.44
1451648_a_at	14276	Folr2	0.003513	0.44
1417292_at	15953	Ifi47	0.015503	0.44
1419609_at	12768	Ccr1	0.016846	0.44
1451939_a_at	51795	Srpx	0.015769	0.44

1418652_at	17329	Cxcl9	0.003514	0.44
1418126_at	20304	Ccl5	0.007603	0.44
1418536_at	15018	H2-Q7	0.023810	0.43
1448756_at	20202	S100a9	0.007228	0.43
1416041_at	20393	Sgk1	0.033619	0.43
1426157_a_at	69165	Cd209b	0.035795	0.43
1420534_at	60596	Gucy1a3	0.013479	0.43
1437502_x_at	12484	Cd24a	0.041641	0.43
1452349_x_at	226695 /// 381308	Ifi205 /// Mnda	0.012127	0.43
1435614_s_at	19417	Rasgrf1	0.047174	0.43
1435330_at	236312	Pyhin1	0.007743	0.43
1449009_at	100039796 /// 21822	Tgtp1 /// Tgtp2	0.012215	0.43
1426183_a_at	170779	Cd209d	0.005509	0.43
1448182_a_at	12484	Cd24a	0.004928	0.43
1460218_at	23833	Cd52	0.003455	0.42
1417065_at	13653	Egr1	0.008469	0.42
1418392_a_at	55932	Gbp3	0.048909	0.42
1436736_x_at	27528	D0H4S114	0.014820	0.42
1423775_s_at	233406	Prc1	0.035006	0.42
1439377_x_at	107995	Cdc20	0.040563	0.42
1422640_at	93880	Pcdhb9	0.031478	0.42
1451206_s_at	227929	Cytip	0.026042	0.42
1419599_s_at	68774	Ms4a6d	0.025037	0.42
1435331_at	236312	Pyhin1	0.032670	0.42
1417936_at	20308	Ccl9	0.003098	0.42
1450839_at	27528	D0H4S114	0.008342	0.42
1424254_at	68713	Ifitm1	0.011765	0.41
1449918_at	70192	Cd209g	0.005493	0.41
1431008_at	110557 /// 68395	H2-Q6 /// LOC68395	0.014877	0.41
1455899_x_at	12702	Socs3	0.023316	0.41
1419940_at	109260	C030018P15Rik	0.034311	0.41
1429947_a_at	58203	Zbp1	0.025885	0.41
1419598_at	68774	Ms4a6d	0.011228	0.40
1449971_a_at	69142	Cd209f	0.028571	0.40
1454694_a_at	21973	Top2a	0.011787	0.40
1428306_at	74747	Ddit4	0.031118	0.39
1452087_at	108670	Epsti1	0.018236	0.39
1424923_at	20715	Serpina3g	0.020444	0.39
1419149_at	18787	Serpine1	0.024408	0.39
1427743_at	---	---	0.035017	0.37
1417793_at	54396	Irgm2	0.032111	0.37
1435477_s_at	14130	Fcgr2b	0.003476	0.37
1448226_at	20135	Rrm2	0.019216	0.37
1438133_a_at	16007	Cyr61	0.012821	0.36
1423100_at	14281	Fos	0.007499	0.36

1423606_at	50706	Postn	0.006657	0.36
1448891_at	80891	Fcrls	0.014829	0.36
1419477_at	93694	Clec2d	0.031739	0.36
1417273_at	27273	Pdk4	0.002911	0.36
1418204_s_at	11629	Aif1	0.011175	0.35
1419882_at	---	---	0.016045	0.35
1425385_a_at	16019	Igh-6	0.019596	0.35
1419043_a_at	60440	Iigp1	0.001927	0.34
1451335_at	231507	Plac8	0.003619	0.34
1417266_at	20305	Ccl6	0.003641	0.34
1420249_s_at	20305	Ccl6	0.012883	0.34
1420141_at	99503	AA517023	0.031367	0.33
1419042_at	60440	Iigp1	0.027297	0.33
1425548_a_at	16988	Lst1	0.024189	0.32
1416039_x_at	16007	Cyr61	0.009270	0.32
1425274_at	65973	Asph	0.003359	0.32
1419513_a_at	13605	Ect2	0.037621	0.32
1418776_at	76074	Gbp8	0.038337	0.32
1418492_at	23893	Grem2	0.015508	0.31
1435716_x_at	20646 /// 84704	Snrpn /// Snurf	0.015181	0.31
1416958_at	353187	Nr1d2	0.023756	0.31
1424270_at	13175	Dclk1	0.041461	0.31
1425470_at	---	---	0.027756	0.31
1449310_at	19217	Ptger2	0.004421	0.31
1420447_at	20860	Sult1e1	0.003591	0.31
1419669_at	19152	Prtn3	0.006908	0.30
1420466_at	20771	Mucl1	0.016056	0.30
1441115_at	52492	D18Ertd232e	0.024330	0.29
1436790_a_at	20666	Sox11	0.029156	0.29
1442531_at	52532	D12Ertd123e	0.011352	0.29
1419762_at	24108	Ubd	0.030878	0.29
1419426_s_at	100038965 /// 100039131 /// 100041593 /// 100042493 /// 100042544 /// 18829 /// 65956	Ccl21a /// Ccl21b /// Ccl21c /// Gm10591 /// Gm13304 /// Gm1987 /// LOC100041593	0.008319	0.28
1425394_at	667597	BC023105	0.026020	0.27
1420582_at	170780	Cd209e	0.031124	0.26
1422860_at	67405	Nts	0.008494	0.25
1426817_at	17345	Mki67	0.011760	0.24
1422411_s_at	100499478 /// 13586 /// 13587 /// 503845 /// 53876	BC151093 /// Ear1 /// Ear12 /// Ear2 /// Ear3	0.003129	0.23
1438009_at	319171 /// 665433	Gm11276 /// Hist1h2ao	0.003065	0.22
1449846_at	13587	Ear2	0.014556	0.21
1422412_x_at	53876	Ear3	0.012386	0.19

1419684_at	20307	Ccl8	0.009257	0.17
1425295_at	93726	Ear11	0.015654	0.12
